# Comparative Pathology of Zoonotic Orthopoxviruses

**DOI:** 10.3390/pathogens11080892

**Published:** 2022-08-09

**Authors:** Amy L. MacNeill

**Affiliations:** Department of Microbiology, Immunology and Pathology, College of Veterinary Medicine and Biomedical Sciences, Colorado State University, Fort Collins, CO 80523, USA; amy.macneill@colostate.edu; Tel.: +1-970-297-5112

**Keywords:** Akhmeta virus, Alaskapox virus, Buffalopox virus, *Camelpox virus*, *Cowpox virus*, Horsepox virus, *Monkeypox virus*, *Vaccinia virus*

## Abstract

This review provides a brief history of the impacts that a human-specific Orthopoxvirus (OPXV), *Variola virus*, had on mankind, recalls how critical vaccination was for the eradication of this disease, and discusses the consequences of discontinuing vaccination against OPXV. One of these consequences is the emergence of zoonotic OPXV diseases, including *Monkeypox virus* (MPXV). The focus of this manuscript is to compare pathology associated with zoonotic OPXV infection in veterinary species and in humans. Efficient recognition of poxvirus lesions and other, more subtle signs of disease in multiple species is critical to prevent further spread of poxvirus infections. Additionally included are a synopsis of the pathology observed in animal models of MPXV infection, the recent spread of MPXV among humans, and a discussion of the potential for this virus to persist in Europe and the Americas.

## 1. Significance of Zoonotic Viruses

The ongoing Coronavirus pandemic has highlighted the serious impacts that contagion can have on human health, behavior, and quality of life. The stressors that pandemics put on the economic and political stability are also profound. Preemptive community awareness campaigns and adequately prepared responses to emerging zoonotic viruses are critical to minimize the negative effects of emerging diseases. Zoonotic viruses, like severe acute respiratory syndrome coronavirus 2 (SARS-CoV-2), have the ability to spread from one or more non-human animal species to humans (*Homo sapiens*). There is significant variability between viruses in mode of transmission, rate of spread, morbidity and mortality caused in different species, and ability to adapt and persist in a population. There are several known zoonotic poxviruses that are being diagnosed in humans more frequently and have emerged in areas of the globe where they are not yet endemic. This review provides descriptions of zoonotic OPXV disease in veterinary species and in humans. This knowledge is critical to minimize the spread of poxviral diseases and understand their potential to emerge in areas of the world where they are not considered endemic.

## 2. Orthopoxvirus Impact on Human History

### 2.1. Smallpox

There is evidence that the causative agent of smallpox, *Variola virus* (VARV), caused mortality in humans for centuries. Egyptian mummies dating from 1186–1070 B.C. appear to have smallpox lesions [1]. Four clinical presentations of smallpox are recognized: ordinary, modified-type, flat-type, and hemorrhagic smallpox. Flat-type smallpox is often fatal in unvaccinated individuals. Hemorrhagic smallpox can be fatal in unvaccinated and vaccinated people alike [2]. There are two variants of VARV; the more deadly strain, VARV major, causes severe disease with up to 30% fatality in unvaccinated people. The VARV minor strain causes morbidity, but the mortality rate following infection is <1% [3,4].

VARV is transmitted via aerosols and fomites. At 7 to 19 days after exposure to VARV, infected people experience fever, chills, lethargy, headache, and back and abdominal pain. A rash typically begins in the mouth and on the face, then spreading to the limbs and trunk [2]. When the rash lesions heal, severe disfigurement is a common sequela [3].

VARV spread throughout Africa, Asia, the Middle East, and Europe over a relatively short period of time. As human population density and trade increased, the prevalence of smallpox rose. It was transported from the Old World to the New World by Spanish explorers, reached the Caribbean in 1507, and was in the Americas by 1520 [3]. As it reached new civilizations, it disrupted trade, led to panic and stress in populations, destroyed economies, and caused the collapse of some societies [3]. In the mid-1700s, approximately 400,000 Europeans died of smallpox each year. It is believed that when smallpox was introduced into the Aztec and Incan Empires, VARV contributed to the deaths of 3–4 million people [5].

### 2.2. Orthopoxvirus Immunization

Following a smallpox outbreak in Boston in 1721, there is documentation that Cotton Mather and Dr. Zabdiel Boylston encouraged smallpox vaccination in the Massachusetts Bay Colony of British America prior to 1726, when they reported their findings at a meeting in London, England. It is said that Mr. Mather read about protection of pox-like lesions in turkeys after healthy birds were exposed to crusts from infected birds, and he may have owned a slave, named Onesimus, who had been immunized as a child [1].

Around the same time period, Lady Mary Montague promoted variolation to protect against smallpox in England. Variolation involves the transfer of material from a smallpox lesion of an infected person to the skin of an uninfected person by scarification. This route of inoculation, rather than the natural route of infection by aerosolization, likely contributed to the success of the practice. Variolation became somewhat accepted after the Princess of Wales agreed to variolate her children [3]. Benjamin Jesty may have used *Cowpox virus* (CPXV) to vaccinate his family against smallpox. However, ultimately, Dr. Edward Jenner was credited with introducing vaccination for smallpox to England in 1796. He collected CPXV from the lesions of a milkmaid and inoculated a boy with the material to protect him against VARV [3]. It is believed that Dr. Jenner later isolated “horsepoxvirus” that ended up being used more extensively to vaccinate people against smallpox in 1817 [6].

Although VACV vaccination was incredibly important in the eradication of smallpox and prevented large numbers of deaths each year, severe complications can be observed in up to 52 out of 1 million people vaccinated. These include progressive vaccinia, generalized vaccinia, vaccinia eczematum, encephalitis, myocarditis, and ocular lesions. Vaccination is not recommended for people with eczema, heart disease, or any type of immunosuppression, as there is evidence these conditions increase the risk of vaccine complications [7].

The VARV vaccines in use today include JYNNEOS^TM^ and ACAM2000^®^. JYNNEOS^TM^ is a non-replicating strain of *Vaccinia virus* (VACV) called Modified Vaccinia Ankara–Bavarian Nordic (MVA-BN). It requires two doses given subcutaneously 28 days apart. ACAM2000^®^ is an attenuated, replication-competent VACV that is administered via scarification. Aventis Pasteur Smallpox Vaccine is an investigational new vaccine that is similar to the ACAM2000 vaccine [8]. In many countries, MVA-based vaccines are preferred in place of traditional smallpox vaccines to protect people against OPXV infections if their risk of exposure to zoonotic OPXV is high. This is because MVA-based vaccines tend to cause fewer side effects than replicating VACV-based vaccines.

### 2.3. Evidence of Orthopoxvirus Exposure in Endemic Areas

Routine smallpox vaccination campaigns were phased out during the early 1970s and 1980s due to successful eradication of the Variola major in 1975 and Variola minor in 1977 [3]. This remarkable success was possible because of the World Health Organization and the biology of VARV. VARV has no animal reservoir, it does not have a latency phase, diagnosis is not difficult, and the vaccine induces a highly protective immune response. Discontinuation of mandatory vaccination programs avoided the risk of complications caused by vaccination. Although smallpox was no longer a disease of concern, the vaccine provided protection against other pathogenic Orthopoxviruses (OPXV) that cause disease in people and are endemic in various regions of the world: *Vaccinia virus* (VACV) in Asia and South America, *Monkeypox virus* (MPXV) in Africa, *Cowpox virus* (CPVX) in Europe, and *Camelpox virus* (CPXV) in the Middle East. Outbreaks, evolution of OPXV genomes, and emergence of zoonotic OPXV could increase transmission and worsen the severity of OPXV diseases [9,10,11]. Early, rapid, and specific diagnosis of both known and emerging OPXV is critical to prevent large outbreaks of OPXV [12].

#### 2.3.1. Seroprevalence in South America

Studies of OPXV prevalence in Brazil detected anti-OPXV antibodies in the sera of 4/688 (<1%) cows, 1/117 (<1%) horses, 1/57 white-eared opossum (*Didelphis albiventris*), and 2/16 common opossum (*Didelphis marsupialis*) [13], as well as 68/269 (25%) tufted capuchin (*Sapajus apella*), 13/27 (48%) black howler (*Alouatta caraya*), 2/12 (17%) South American coati (*Nasua nasua*), and 1/20 (5%) agouti (genus *Dasyprocta*) [14]. VACV neutralizing antibodies were detected in sera from 35/184 (19%) dogs (*Canis familiaris*) and 13/90 (14%) South American coati in another study. No clinical signs of OPXV infection were described in dogs or coati [15]. Although no antibodies were found in rodent sera (n = 103) in one study, VACV DNA was detected in rodent feces [6/115 (5.2%)] and urine [1/55 (1.8%)] [16]. Anti-OPXV antibodies were found in 53.1% of vaccinated and 6.1% of non-vaccinated people tested in Brazil (n = 240). None of the people tested reported symptoms of poxviral disease [17]. Based upon the increase in the number of publications reporting OPXV infections in humans, the incidence of symptomatic human OPXV infection has increased dramatically in the past 20 years. This is likely due to an increase in the number of people who have not been vaccinated against smallpox and so lack protective anti-OPXV antibodies [18].

#### 2.3.2. Seroprevalence in Africa

Anti-OPXV antibodies were detected in 33.3% shrews, 14.7% rodents, and 2.1% primates sampled in Zambia [19]. In the Democratic Republic of the Congo, anti-OPXV IgG was found in two rope squirrels (*Funisciurus* spp.) and in one animal from each of the following species: Lorrain dormouse (*Graphiurus lorraineus*), Emin’s pouched rat (*Cricetomys emini*), sun squirrel (*Heliosciurus* sp.), common rufous-nosed rat (*Oenomys hypoxanthus*), and four-toed elephant shrew (*Petrodromus tetradactylus*) [20].

#### 2.3.3. Seroprevalence in Europe

In Hungary, between 2011 and 2012, 286/1587 (18%) of the rodents tested were positive for anti-OPXV antibodies. The highest seroprevalence was seen in bank voles (*Myodes glareolus*), followed by striped field mice (*Apodemus agrarius*), wood mice (*Apodemus sylvaticus*), and yellow-necked mice (*Apodemus flavicollis*) [21]. In a survey of 962 animals (15 species of rodents), the seroprevalence of anti-OPXV antibodies was calculated to be 33% in Finland, 32% in Germany, and 3.2% in Siberia [22]. OPXV seropositivity was observed in 16.5% of Finnish field vole (*Microtus agrestis*) samples collected between 2008 and 2010 (n = 709) [23]. Anti-OPXV antibodies have been detected in German bank voles, common voles (*Microtus arvalis*), yellow-necked mice, and wood mice. In Germany, anti-OPXV antibodies were most commonly detected in bank voles, but seroprevalence in all species tested varied significantly in different areas of the country [24].

#### 2.3.4. Seroprevalence in the Middle East

The seroprevalence of anti-OPXV antibodies in animal populations in the Middle East is not well described. Camelpox virus seems to have a restricted host range as compared to other zoonotic OPXVs.

### 2.4. Potential for Orthopoxvirus Bioterrorism

Historically, smallpox has been used as a bioweapon. In 1763, the English purposefully donated blankets from a smallpox ward to North American Indians who were fighting against them with the French [3]. Given the severe consequences of the spread of smallpox through the human population, the zoonotic potential of many OPXV, the ability of OPXV to spread via respiratory droplets, and the lack of antibody protection in the vast majority of humans, the potential for the development of an OPXV as a bioweapon is considered high [25]. As such, animal models of smallpox have been developed to understand the disease in more detail, guard against alterations of OPXV that could advance their potential as bioweapons, and develop safe and effective vaccines and treatments for both weaponized poxviruses and naturally emerging OPXVs. An excellent discussion of animal models of OPXV was published in 2010 [26].

## 3. Vaccinia virus

*Vaccinia virus* (VACV) is the OPXV that was used to vaccinate people throughout the world against smallpox. Extensive research has been conducted using VACV infection in animal models to study OPXV biology and understand VARV pathogenesis in humans. There are several variants of VACV that have been named according to the species or location they were first identified in. These include Rabbitpox virus (RPXV, New York 1932 and Utrecht 1941), Buffalopox virus (BPXV, northern India 1967), Horsepox virus (HSPV, Mongolia 1976), and Brazilian VACV (Brazil 1999) [27,28]. Different variants of VACV have variable pathogenicity in different species. This is likely due to alterations in gene expression by the variants. For example, HSPV is known to encode genes that are not expressed by other VACV variants [29]. Phylogenetic analysis of poxviruses suggests that HSPV and RPXV share a viral ancestor that separates them from other VACV variants [30].

VACV is considered endemic in areas of South America and Asia. The virus causes disease in several animals (Table 1), including rodents, cattle, horses, monkeys, and humans [31]. Experimental transmission of VACV from cow feces to mice has been documented. No signs of poxviral disease were observed in the mice; however, anti-OPXV antibodies were detected after mice were exposed to cow feces containing VACV DNA [32]. The number of human cases of VACV infection continues to increase as the number of people vaccinated against OPXV decreases.

### 3.1. Vaccinia virus in South America

VACV infections in humans were first reported in Brazil in 1999. There are two genetically different groups of VACV circulating in Brazil. The two groups of VACV grow differently in BSC-40 cell cultures; Group 1 Brazilian VACVs form large viral plaques, while Group 2 Brazilian VACVs form small plaques. Group 1 Brazilian VACVs lack the viral A56R gene and are typically less virulent [89,90]. B-type viral inclusions are often detected within skin lesions of affected veterinary species and humans [91].

Rodents are a likely reservoir for VACV. High titers of virus were isolated from the peritoneum and testicles of a non-symptomatic house mouse (*Mus musculus*) during a 2005 outbreak in Mariana County [77]. VACV DNA was found in 6.4% of rodents tested, and anti-OPXV antibodies were found in 10.5% of animal sera collected. Overall, 25.7% of the rodents and 7.6% of the marsupials tested were considered positive for exposure to VACV [92].

The incubation period for VACV in cows is 1–7 days. VACV-infected cows present with exanthematous lesions on their teats and udders. These can be extensive, bloody, and painful. Nursing calves can develop lesions on their lips and nose [31]. Milk production is decreased, particularly in cows that have secondary mastitis [89]. Milk can contain VACV particles [93,94].

Horses with VACV infection often present with painful lesions on the lips and nose. Udders can also be affected. Nursing foals can have lesions in their mouths. The lesions are proliferative or papular and may ulcerate. Lesions typically last 6–12 days before healing [31].

The incubation period for VACV in humans is approximately 3–5 days. Active disease symptoms usually last 4 weeks; however, secondary bacterial infections can prolong the disease course. Humans infected with VACV can experience fever, headache, and malaise for 2–5 days. Lesions are usually first observed on the hands and arms [31,89,95]. The lesions may spread to the mouth, scrotum, and/or vulva and are often itchy. Initially, lesions appear as papules that develop into pustules, then become necrotic and ulcerated over approximately 12 days. Eventually, the lesions scab over. Lymphadenopathy is common. Less common signs of disease include anorexia, dehydration, nausea, and sweating [31]. Decreases in T-cell activation have been documented in people infected with Brazilian VACV [96,97]. Downregulation of co-stimulatory molecules (CD80/86) on B-cells and CD14^+^ cells was reported [97]. Stimulation of peripheral blood mononuclear cells by VACV appears variable and may depend on the technique or timing of sample collection following infection [96,97]. There is strong evidence that immunosuppressed people can experience a more severe disease course after zoonotic transmission of VACV, just as they can following vaccination [91].

### 3.2. Vaccinia virus in Asia

Buffalopox virus (BPXV) is the most common strain of VACV infection reported in humans in India. Outbreaks have occurred in Bangladesh, Egypt, India, Indonesia, Iran, Nepal, and Pakistan [98]. In buffalo (*Bubalus* spp.) and cows (*Bos taurus*), it causes lesions on teats and udders, inguinal area, ears, and eyelids [98,99,100,101]. Decreased milk production is observed with or without secondary mastitis [99,100]. Between 1992 and 1996, buffalo and humans from Maharashtra State had evidence of BPXV infection. Manifestations of the disease included cases in children with disseminated pox lesions on their face, arms, and rump [101]. Another outbreak occurred in Maharashtra State in 2008 and 2009 that involved 351 humans and several buffalo. Attack rates of 6.6% in humans and 11.7% in buffalo were reported. Most cases in humans occurred in males 20–59 years of age. Disease progression in humans is similar to that seen with other subtypes of VACV, including fever; malaise; painful skin lesions on the hands, feet, face, and mouth; and lymphadenopathy [99,100]. In a 2017 outbreak in Moinabad, skin lesions in humans took up to 6 weeks to scab over [102].

In the Punjab province of Pakistan, buffalo cows with wart-like nodules and inflammatory skin lesions on their teats and udders were tested for BPXV. PCR detected VACV in 49 of the 63 scabs collected. Positive animals had peripheral blood neutrophilia and lymphocytosis [103].

## 4. Monkeypox virus

*Monkeypox virus* (MPXV) was first described as pox-like outbreaks in two groups of cynomolgus monkeys (*Macaca fascicularis*) at the State Serum Institute in Copenhagen, Denmark. A total of 20 to 30% of monkeys housed together were affected by a rash that progressed to papules, which covered the trunk, tail, face, limbs, hands, and feet. Lesions ulcerated, then formed crusts, which fell off, leaving scars after a period of two to four months. The animals were healthy otherwise, and no lesions were observed in internal organs at necropsy [104]. The first human MPXV infection was reported in 1970 [72]. There are two clades of MPXV that are genetically distinct: one isolated in West Africa and one in the Congo Basin. The Congo Basin MPXV is more virulent than the west African MPXV [105]. The fatality rate associated with the Congo Basin MPXV is estimated to be 10.6%, while the West African MPXV is less than 3.6%. MPXV genomes detected in humans during the 2022 outbreak belong to the West African clade [106]. Transmission of MPXV occurs via contact with skin lesions and respiratory droplets. The disease can also be spread through scratches and bite wounds from infected animals [107,108]. Human-to-human transmission has been well documented [60,109,110,111,112,113,114]. The incubation period (between infection and onset of clinical disease) is estimated to be 7–18 days in humans [115,116,117].

### 4.1. Clinical Signs

There is evidence of asymptomatic OPXV infection (presumed to be caused by MPXV) in both vaccinated and unvaccinated people in Ghana [118]. If asymptomatic individuals shed MPXV in oropharyngeal (or other) secretions, they could contribute to inadvertent human-to-human spread of the disease. Classic symptoms of MPXV include fever, pruritis, skin and mucocutaneous lesions, malaise, headache, backache, and lymphadenopathy. Often, disease symptoms are more severe in unvaccinated individuals [119,120]. Disease sequalae observed in unvaccinated people during an outbreak in Zaire (1980–1985) included secondary bacterial infections in 19% unvaccinated people, bronchopneumonia (12%), vomiting and diarrhea leading to dehydration (7%), corneal ulceration (4%), septicemia (<1%), encephalitis (<1%), and death (11%) [120]. In a study of a human outbreak of Congo Basin MPXV in the Kivu provinces (2011–2015), the fever lasted 1–4 days, and skin lesions began as macules that progressed to papules, vesicles, pustules, then crusts, and scarred 14–24 days after macules were detected [115]. Lesions can occur all over the body, including the mouth and genitalia [18]. The timing of disease was slightly different in an outbreak of West African MPXV in the United States (2003). The fever lasted 2–13 days, and the rash lasted 7–24 days [117]. The timing of the onset of fever and development of a rash differed between individuals who were scratched or bitten by an infected animal and individuals who simply handled infected animals [108]. Oropharyngeal lesions occurred in 76% of unvaccinated people as compared to 47% of vaccinated people [119]. Virus replication within lesions in the upper respiratory tract likely contributes to spread of the virus in respiratory droplets. Lymphadenopathy is an important feature of MPXV infection that is not observed during VARV infection (smallpox). Lymphadenopathy begins 1–2 days before the onset of the rash and occurs in approximately 85–90% of unvaccinated people infected with MPXV [18,119,121,122]. Coughing and/or conjunctivitis occur in ≥30% of unvaccinated people [119].

The median age of humans infected with MPXV in the Democratic Republic of the Congo between 1996 and 1997 was 10 years old, and disease was slightly more common in men (56.8%) [60]. A review of 1057 confirmed MPXV cases in the Democratic Republic of the Congo between 2011 and 2015 indicated that approximately 60% of cases were men and that most were between 10 and 19 years old [122]. A study of an outbreak of 122 cases in Nigeria in 2017 found that the median age of affected individuals was 29 years old, and 69% of cases occurred in males [123]. In a past outbreak, the incidence of cases in unvaccinated people was over 2.5× greater than in vaccinated people [122]. More recently, it has been estimated that 80–90% of cases of human MPXV are diagnosed in unvaccinated people [124]. In people who have not been vaccinated for smallpox, case fatality rates between 9.8% and 13% have been reported [18,119,120,121]. Deaths occur more often in the very young and in immunosuppressed people [111,119,120]

Natural infection with MPXV has been detected in many species (Table 1) [67]. The first non-captive species that MPXV was diagnosed in was a Thomas’s rope squirrel (*Funisciurus anerthrus*) [63]. Fatal MPXV infection was described in an infant sooty mangabey (*Cercocebus atys*) from the Ivory Coast. The monkey was found dead with disseminated red crusts on its extremities and abdomen. Eosinophilic cytoplasmic viral inclusion bodies and severe secondary bacterial infection were observed in histologic sections of skin. MPXV DNA was isolated from several organs [68]. Disease in wild chimpanzees (*Pan troglodytes*) at the Taï National Park in the Ivory Coast was described in three infant chimpanzees, one of whom died. The chimpanzees had a cutaneous rash, coughing, and respiratory distress. MPXV DNA was detected in 62/492 (12.6%) fecal, 11/55 (20%) urine, and 5/26 (19.2%) fruit wedge samples tested. MPXV DNA was also detected in maggots and materials around the individual that died from disease [125].

### 4.2. Pathology

There are very few reports of alterations in complete blood counts and serum biochemistry profiles of people infected with MPXV, but leukocytosis (45% of patients), thrombocytopenia (35%), hypoalbuminemia (50%), decreased urea nitrogen concentration (61%), and increased alanine aminotransferase and aspartate aminotransferase have been documented [117]. The combination of increased aminotransferases and decreased albumin and urea nitrogen is highly suggestive of liver damage and decreased liver function. Experimental infection of cynomolgus monkeys with very high doses of aerosolized MPXV-Zaire caused anemia, leukocytosis, hypoalbuminemia, increased liver enzymes, and azotemia [126,127].

Histopathologic findings in MPVX-infected skin lesions are remarkably similar in humans and cynomolgus monkeys. Intraepidermal vesicles and pustules can be observed. As lesions progress, they become ulcerated and form crusts with marked suppurative inflammation within and below the crusts. Around the crust, there is significant epidermal hyperplasia and ballooning degeneration of keratinocytes associated with a ring of edema and mononuclear inflammation (macrophages and lymphocytes) that extends into the dermis. Keratinocytes often contain intracytoplasmic inclusion bodies [117,126].

### 4.3. Experimental Infection

Guinea pigs (*Cavia porcellus*), golden hamsters (*Mesocricetus auratus*), European rabbits (*Oryctolagus cuniculus*), and most laboratory mice are relatively resistant to MPXV infection [67,128]. Experimental infection of 38 strains of mice found CAST/EiJ, PERA/EiJ, and MOLF/EiJ mice susceptible, likely because they are immunodeficient [129]. Neither Balb/c nor C57BL/6 mice showed signs of disease after intradermal (i.d.) or intranasal (i.n.) inoculation with 10^5^ pfu MPXV-west African. Mild disease was observed in Balb/c mice but not C57BL/6 mice, similarly inoculated with MPXV-Zaire [130]. This is one of the reasons that other experimental models of MPXV have been investigated. More critically, it is important to know what the disease looks like in: (1) species that are naturally susceptible to MPXV infection, (2) species that cohabitate with humans living in areas where MPXV is endemic, and (3) species that may become reservoirs of MYXV if the disease spreads to other regions of the world.

Aerosolized MPXV infection of cynomolgus monkeys given ≥10,000 plaque-forming units (pfu) of MPXV-Zaire caused viral replication and, subsequently, severe inflammation and necrosis throughout the body. Exanthema, enanthema, fibronecrotic bronchopneumonia, pleuritis, inflammation and necrosis throughout the intestinal tract, necrotizing lymphadenopathy, splenitis, necrotizing hepatitis, orchitis, and oophoritis were all reported [126,127].

Kellen’s dormouse (*Graphiurus kelleni*) was tested for susceptibility to MPXV infection. Intradermal and i.n. inoculation caused loss of body weight, decreased activity, hunched posture, unkempt haircoats, dehydration, conjunctivitis, and death in most animals tested. Symptom severity was dose dependent with 100% mortality at 200 pfu, 63% at 20 pfu, and 0% at 2 pfu following i.n. inoculation. The virus was detected in nasal lavage fluid as early as 2 days post-inoculation (dpi) and in several organs (including lung) at 4 dpi. Necropsy findings were somewhat different from those in cynomolgus monkeys that were given far higher doses of MPXV. Similarities included necrotizing lymphadenopathy and necrotizing hepatitis. In the dormice, necrosis was also detected in the spleen, thymus, and bone marrow. The lungs, intestinal tract, gall bladder, and brain had hemorrhagic lesions. Additionally, rhinitis with syncytial cell formation and B-type viral inclusions were reported [58].

Black-tailed prairie dogs (*Cynomys ludovicianus*) have been experimentally infected i.n. with 10,000 pfu MPXV-US. The animals (n = 3) had to be euthanized within 16 dpi. All had skin and/or oral lesions. Virus replication was detected in the lymph nodes at 5 dpi. Histologically, they had necrosis in skin lesions and lymphoid tissues. One prairie dog was co-housed with an experimentally infected animal and contracted the disease. Lymphoplasmacytic interstitial pneumonia was documented, although two of the prairie dogs also had lung mites [131]. At 6 × 10^3^ pfu MPXV i.n., the signs of disease included weight loss and development of skin lesions. Serum biochemical abnormalities (including increased alkaline phosphatase, alanine transferase, glucose, and globulins, as well as decreased albumin) were reported and correlated with evidence of hepatic necrosis. Transmission of the virus was observed in animals that shared bedding or were co-housed with infected prairie dogs [132].

Inoculation of Thomas’s rope squirrel (*Funisciurus anerythrus*) with 10^6^ pfu MYXV-Zaire i.n. caused slightly less fatality than that observed in prairie dogs infected with MPXV-US (75% as compared to 100%). Clinical signs included ocular and oral lesions seen 6 and 8 dpi, respectively, tachypnea 9 dpi, and skin lesions 11 dpi. A sentinel animal became infected and was tachypneic. Histopathology of skin lesions of animals infected i.n. were similar to those reported in other species with epidermal necrosis and ulceration, suppurative inflammation within and under ulcers, and surrounding epidermal spongiosis and edema associated with mononuclear inflammation. Reactive endothelium and fibrin deposition, which is noted in several other poxvirus lesions, were also described. Half of the animals inoculated by scarification or i.d. injection had to be euthanized, but these animals developed skin lesions much sooner (3 dpi). However, histopathology results suggested that their lesions were less severe with epithelial hyperplasia and lymphoplasmacytic vasculitis. Lymphoplasmacytic interstitial pneumonia and lymphoid hyperplasia were also noted. Low numbers of animals had necrosis in lymph nodes, a finding that seems to be common in other species. The virus was detected in oral secretions from all animals from 3 dpi up to 25 dpi [133].

Intranasal or intraperitoneal infection of thirteen-lined ground squirrels (*Ictidomys tridecemlineatus*) with 10^5.1^ pfu MPXV-west African US caused anorexia and lethargy but no other signs of disease. However, all animals died between 6 and 9 dpi. Hepatic and splenic necrosis and consolidation of lung tissue were observed in i.p.-inoculated animals. Intranasal inoculation caused multifocal steatosis of the liver, interstitial pneumonia, and necrosis in some lymph nodes. Basophilic viral inclusions were observed in hepatocytes. Immunohistochemistry indicated viral antigen was also present in mesothelial cells and adipocytes of some animals. The virus was isolated from blood, oral swabs, and all organs tested [134].

Gambian pouched rats (*Cricetomys gambianus*) were inoculated with 4 × 10^4^ pfu MPXV by scarification [135] or with very high doses (10^6^ pfu) of MPXV i.d. or i.n. [136]. Fatality occurred in one rat infected i.d., but no signs of disease were observed in a sentinel rat exposed to the infected animals. However, significant amounts of virus were detected in saliva of the i.d.- and i.n.-infected animals [136].

### 4.4. Epidemiology

In 2014 and 2016, outbreaks of MPXV were observed in captive chimpanzees in Cameroon, but no symptoms were reported in humans. In 2018, 63 people who worked with the chimpanzees or lived in the nearby village were tested for exposure to OPXV. These people were born after 1980 (after mandatory smallpox vaccinations were discontinued). Serum anti-OPXV IgG was detected in 6.3% and IgM in 1.6% of these individuals, indicating they had been exposed to an OPXV [118]. A study that surveyed 939 people in the Democratic Republic of the Congo identified age (≥18 years-old), sex (male), and occupation (hunter) as potential risk factors for contracting MPXV [137]. A review of 71 documents indicated that MPXV outbreaks occur more commonly in men, people <30 years old, people with animal contact, and in rural areas [138].

When human infections are identified, animals in and around human settlements are often tested for MPXV. Serum anti-OPXV antibodies have been detected in red-legged sun squirrel (Heliosciurus rufobrachium)*,* Congo rope squirrel (*Funisciurus congicus*), Thomas’s rope squirrel (*Funisciurus anerythrus*), Emin’s pouched rat (*Cricetomys emini*), four-toed elephant shrew (*Petrodromus tetradactylus*), and wild boar (*Sus scrofa*) [60,139]. There are reports of anti-OPXV antibodies in sera from monkeys, squirrels, rodents, birds, and antelope [121]. The percentage of animals that are positive for anti-OPXV antibodies ranges from 2/68 (2.9%) [121] to 15/59 (25.4%) [60]. Specific radioimmunoassays or immunofluorescence testing for anti-MPXV antibodies suggest that infection can occur in red-tailed monkey (*Cercopithecus ascanius*), Allen’s swamp monkey (*Allenopithecus nigroviridis*), lesser spot-nosed monkey (*Cercopithecus petaurista*), and, possibly, western red colobus (*Piliocolobus badius*) [121]. Table 1 provides a list of species reported to have acquired MPXV infection in nature.

Between 1970 and 1983, 80% of human MPXV cases (n = 155) occurred in Zaire [121]. In 2003, 37 people were diagnosed with and 10 people were suspected of having MPXV infection in the United States [108]. Several species of animals imported from Ghana had been housed with prairie dogs. Disease symptoms in prairie dogs (including watery eyes, congestion, and skin lesions) were noted after distribution of several animals to private households. Humans became ill within 4–21 days of direct contact with the prairie dogs. Histopathology of skin lesions was typical of poxvirus infection [140]. In the Democratic Republic of the Congo, the incidence of MPXV infection was 0.64 cases/100,000 people in 2001. In 2013, case incidence rose to 2.82/100,000 people [124]. It is clear that the disease is becoming more common in humans, likely due to the lack of protection from OPXV that previously had been provided by widespread smallpox vaccination.

Over 120 cases of human MPXV were reported in countries outside of Africa in the week before 20 May 2022 [141]. As of 27 June 2022, 3413 human MPXV infections have been reported across >50 countries in Europe, Latin America, the Middle East, North America, and Asia [142]. Many of the cases are associated with men traveling by plane from Nigeria to other countries [143,144,145]. At least two people coming from Nigeria infected with MPXV stopped in Canada before traveling to the United Kingdom and the United States [146]. Many of these cases are occurring in men who likely have had direct contact with lesions during sex or indirect contact via fomites [147].

## 5. Cowpox virus

*Cowpox virus* (CPXV) causes disease in many mammalian species (Table 1). Orthopoxviruses that are classified as CPXV can be separated into at least four separate clades using phylogenetic analysis: VARV-like, VACV-like, CPXV-like-1, and CPXV-like-2 clades [148]. Naturally acquired CPVX infections of mice, gerbils, voles, rats, cattle, cats, dogs, horses, primates, and several animals housed in zoos have been described [24,149,150]. There are several incidents of transmission of CPXV from cats to humans [151,152,153,154] and rats to humans [40,155]. Transmission of CPVX from a brown rat (*Rattus norvegicus*) to an Asian elephant (*Elephas maximus*) to a human has also occurred [156].

CPXV lesions appear similar to those caused by other OPXVs; however, the infected cells contain eosinophilic A-type (Cowdry) viral inclusions as opposed to the more basophilic B-type inclusions usually observed with other OPXVs. The virus also forms red, hemorrhagic pocks on chicken chorioallantoic membranes, rather than inflammatory, white pocks that are observed with other OPXVs.

Short-tailed field voles (Microtus agrestis) are likely a reservoir for CPXV. A negative correlation between CPXV infection and survival has been reported in this species in England [157]. Likewise, common voles (*Microtus arvalis*) can be infected with CPXV. Following experimental oronasal inoculation with 10^4^ pfu of CPXV isolated from a wild common vole, skin lesions in experimentally infected voles were not observed, but virus was shed in oropharyngeal secretions 4–8 days after inoculation [158]. Bank voles (*Myodes glareolus*) inoculated by the oronasal route with CPXV show no clinical signs of disease and do not shed detectable amounts of virus, but they do seroconvert. Seroconversion was also observed in low numbers of voles that were in contact with the inoculated voles [159]. In a study of bank voles collected from 2017–2018 in Germany, CPXV DNA was detected in 5/509 (0.98%) of nasal septum samples, and virus was isolated from one animal [160]. In France, montane water voles (*Arvicola scherman*) are reported to have a CPXV seroprevalence of 41.8% (n = 158) [161].

Rats are known to acquire natural infections with CPVX. Experimental infection of Wistar rats and fancy rats (*Rattus norvegicus domestica*) via i.n. or i.n. and scarification with 10^6^ pfu of CPXV causes disease within 11 days [162]. Disease signs in rats include ulcerative skin lesions, unkempt fur, sneezing, ocular discharge, dyspnea, anorexia, and lethargy [162,163,164,165]. Pneumonia and abdominal distention were observed in some studies [164,165]. In one study, histologic sections showed necrotizing lesions in the skin and upper respiratory tract with eosinophilic cytoplasmic inclusion bodies. Some rats had bronchopneumonia, pulmonary edema, emphysema, and/or meningoencephalitis. Rats that were in contact with the infected animals developed cutaneous lesions on day 10 but had a milder disease course and recovered from CPXV infection within 36 days [163]. Rats have been shown to shed virus as early as 5 days and up to 21 days after exposure [162].

Cats likely acquire CPXV from infected rats [166]. Cats with CPXV infection often present with skin lesions on the head, neck, and forelimbs [149]. Conjunctiva and mucous membranes can also be affected [153]. Lesions begin as erythematous macules or papules that develop into nodules, which ulcerate and eventually crust over within 4–5 weeks. Histologically, a necrotizing dermatitis with ballooning degeneration of epithelial cells is observed. Lesions can become secondarily infected with bacteria. Lymphadenopathy has been reported [153]. Occasionally, severe systemic infection or necrotizing pneumonia occurs, which can be fatal. Viral inclusions can be observed in bronchoalveolar lavage samples from some cats with CPXV pneumonia [167]. Fatalities are more common in young kittens and immunosuppressed adults [166]. The OPXV seropositivity in 226 cats tested in Italy was 33.3% [154]. Evidence for the spread of CPXV via fomites has been documented [149].

CPXV can cause severe disease in many species housed in zoos. In Germany, MVA vaccination is used to protect zoo animals from CPXV infection [48]. Fatal CPXV infections can occur in banded mongooses (*Mungos mungo*) [44,48] and jaguarundi (*Herpailurus yagouaroundi*) [48]. Infected mongooses were anorexic, lethargic, and dyspneic. Imbalance, petechia, pox-like skin lesions, mucosal ulcers, and lymphadenopathy were observed. Histopathology revealed necrotizing and inflammatory lesions in lymph nodes, skin, liver, and spleen. A brown rat in the enclosure also had skin lesions consistent with OPXV infection [44]. As expected, CPXV infection severity varies between species. Four cotton-top tamarins (*Saguinus oedipus*) housed in an animal park in Germany died from CPXV infection in 2010. The animals had alopecia and some ulcerations of skin and mucocutaneous junctions. CPXV was isolated from the skin of these animals. CPXV DNA was detected by PCR in oral swabs from common marmosets that were housed next to them. The marmosets showed no clinical signs of disease [168]. There are many other examples of CPXV infection in zoo animals. CPXV DNA was isolated from a giant anteater (*Myrmecophaga tridactyla*) that was anorexic, lethargic, and had vesicular and ulcerated skin lesions on its nose, mouth, vulva, flanks, and feet. Clinical pathology abnormalities included neutrophilia with a left shift, hypoglobulinemia, and increased alkaline phosphatase. The anteater survived infection with supportive care [169]. CPXV infection of Patagonian maras (*Dolichotis patagonum*) was detected in captive animals in the Netherlands. Animals had conjunctivitis and were anorexic. At necropsy, ulcers were observed on the tongue and buccal mucosa, and petechial hemorrhages were noted on the heart. Histopathology indicated there was lymph node and splenic lymphoid hyperplasia, renal tubular necrosis, and pulmonary edema. CPXV DNA was isolated from these animals [42].

Hoofstock are also susceptible to CPXV infection. In cattle, CPXV causes ulcerated lesions on the udder and teats. Lesions can become generalized. The disease course typically lasts 4 weeks [170]. CPXV can cause abortion and premature birth in horses. The mares are typically asymptomatic, but papules on the skin and ulcers in the mouth that contain A-type inclusion bodies can be detected in the foals [171,172]. CPXV disease in alpaca ranged in severity from localized lesions to fatalities. Seroprevalence varied from 16.1% to 81.2% in four herds that were affected by the outbreak. CPXV DNA and anti-OPXV antibodies were found in common voles, striped field mice, and bank voles captured at the alpaca farms [173].

A localized skin lesion with fatigue, fever, and lymphadenopathy are often observed in humans infected with CPXV, but generalized CPXV infections have been reported. The generalized disease causes ulcerated nodules in several areas of the body, sometimes including the groin. Skin lesions typically heal within 2–4 weeks, unless secondary bacterial infection occurs [174]. The disease can be fatal in immunosuppressed people [175].

## 6. Camelpox virus

In dromedary camels (*Camelus dromedarius*) and Bactrian camels (*C. bactrianus*), *Camelpox virus* (CMLV) can cause fever; nasal discharge; pox on the head, neck, throat, lips, extremities, inguinal and perianal areas, and scrotum; respiratory stress; and gastrointestinal upset [37,176]. Morbidity is often low (1.1%), and most affected camels are >2 years old [176]. However, fatality rates of 12–25% have been reported [37,176]. Histologically, skin lesions have dermatitis with ballooning of keratinocytes. Less commonly, lymphoid hyperplasia is observed. Bronchitis or pneumonia can occur in animals that succumb to disease [176]. A human outbreak of CMLV in Rajasthan, India, occurred in three people in 2009. People reported fever, itching, erythema, and edema that developed into classic poxvirus lesions on the hands, including vesicles that ulcerated approximately 7 days and formed crusts approximately 15 days after signs of disease began. Virus DNA was isolated from the lesions on one person. Virus was isolated from 14 dromedary camels associated with the outbreak [37]. Camel-to-human transmission of CMLV was also reported in a 2014 outbreak of poxvirus infection in eastern Sudan. Humans experienced fever, malaise, itching, erythema that progressed to nodules 7–10 days after contact with affected camels, and clearance of skin lesions 30–40 days after the onset of disease. Viral DNA was detected in two dromedary camels and three out of four humans tested. Virus was isolated from one of the affected people [177].

## 7. Unclassified, Genetically Distinct Orthopoxviruses

### 7.1. Alaskapox virus

In 2015, an Alaskan woman presented with a pox lesion on her shoulder and lymphadenopathy. She reported 5 days of fever, malaise, and fatigue. The lesion took 6 months to heal. It was unclear if the disease was transmitted through contact with a human or indigenous small mammals [178]. Genetic testing indicated the virus is a unique OPXV [35].

### 7.2. Akhmeta virus

In 2013, genetically distinct OPXV DNA was isolated from the skin lesions of two Georgian milkers with symptoms of fever, eschars, hand swelling, and lymphadenopathy. Cows, rodents, and shrews in the area tested positive for anti-OPXV antibodies [34].

## 8. Discussion

Zoonotic OPXV are endemic in specific regions of the world. In the past, they were rarely diagnosed in humans, and outbreaks were relatively easy to contain. This seems to be changing due to the lack of protective immunity in the human population following cessation of smallpox vaccination. It is possible that zoonotic OPXV will become endemic in new areas of the globe due to human spread of the diseases into native populations of animals that are susceptible to the viruses. This necessitates that OPXV diseases are recognized rapidly in non-human species that are in contact with people and can contribute to spread of the disease. Table 2 provides a summary of pathology observed in key species affected by zoonotic OPXV. Disease signs are quite similar in most species, but minimal disease severity in some species can make diagnosis difficult. Additionally, it is nearly certain that additional species are infected by zoonotic OPXVs, including bats [179,180]. Surveillance and reporting of OPXV pathology in new species will be important to help monitor the spread of OPXV diseases. Given the large number of species that MPXV is known to infect, it is likely to become endemic outside of Africa if the disease is not contained.

Isolation of the affected individuals and ring vaccination of people in contact with contagious animals and people are currently the best ways to prevent the spread of zoonotic OPXV diseases. The ability of countries to respond to OPXV infections rapidly and effectively has been tested by the MPXV outbreaks occurring throughout the globe. The cooperation of countries with the World Health Organization and strong leadership to coordinate public health agencies will be critical to manage OPXV, as it was during the campaign to eradicate smallpox. Continued advancement of our understanding of OPXV biology and pathology in different species is needed to protect people and animals from expansion of zoonotic OPXV disease.

## Figures and Tables

**Table 1:** Orthopoxvirus taxonomy and species naturally affected.

Virus	Selected Variants or Clades	Species Susceptibility to Natural Infection [Reference]
Abatino macaquepox ^1^		Tonkean macaque (*Macaca tonkeana*) [33]
Akhmeta ^1^		Cattle (*Bos* spp.) [34] Human (*Homo sapien*) [34]
Alaskapox ^1^		Human (*Homo sapien*) [35]
Ectromelia		Mice (*Mus* spp.) [36]
Camelpox		Dromedary camel (*Camelus dromedarius*) [37] Bactrian camel (*Camelus bactrianus*) [37] Human (*Homo sapien*) [37]
Cowpox	Brighton red	Laboratory strain
	Calpox	Common marmoset (*Callithrix jacchus*) [38]
		Wood mice (*Apodemus sylvaticus*) [39] Yellow-necked mice (*Apodemus flavicollis*) [24] Striped field mouse (*Apodemus agrarius*) [21] Chinese striped hamster (*Cricetulus barabensis*) [22] Bank vole (*Myodes glareolus*) [39] Gray red-backed vole (*Myodes rufocanus*) [22] Short-tailed field vole (*Microtus agrestis*) [39] Common voles (*Microtus arvalis*) [24] Tundra vole (*Microtus oeconomus*) [22] Reed vole (*Microtus fortis*) [22] European water vole (*Arvicola amphibius*) [24] Brown rat (*Rattus norvegicus*) [40] Great gerbil (*Rhombomys opimus*) [27] Libyan jird (*Meriones lybicus*) [41] Patagonian mara (*Dolichotis patagonum*) [42] North American beaver (*Castor canadensis*) [43] Banded mongoose (*Mungos mungo*) [44] Red fox (*Vulpes vulpes*) [45] Red panda (*Aiulurus fulgens*) [43] Cat (*Felis catus*) [46] Ocelot (*Leopardus pardalis*) [47] Cheetah (*Acinonyx jubatus*) [47] Lion (*Panthera leo*) [47] Sumatran tiger (*Panthera tigris sumatrae*) [45] Leopard (Panthera pardus) [47] Jaguar (*Panthera onca*) [47] Cougar (*Puma concolor*) [47] Jaguarundi (*Herpailurus yagouaroundi*) [48] Giant anteater (*Myrmecophaga tridactyla*) [47] Malayan tapir (*Tapirus indicus*) [49] Black rhinoceros (*Diceros bicornis*) [27] Southern white rhinoceros (*Ceratotherium simum simum*) [50] Asian elephant (*Elephas maximus*) [50] African bush elephant (*Loxodonta africana*) [27] Okapi (*Okapia johnstoni*) [50] Llama (*Lama glama*) [51] Alpaca (*Lama pacos*) [52] Wild boar (*Sus scrofa*) [53] Cattle (*Bos* spp.) [54] Horse (*Equus ferus caballus*) [55] Barbary macaque (*Macaca sylvanus*) [56] Human (*Homo sapien*) [57]
Monkeypox	Copenhagen Congo Basin (Zaire) West African West African (USA)	Kellen’s dormouse (*Graphiurus kelleni*) [58] Lorrain dormouse (*Graphiurus lorraineus*) [59] Jerboas (*Jaculus* spp.) [59] Emin’s pouched rat (*Cricetomys emini*) [27] Gambian pouched rat (*Cricetomys gambianus*) [59] Common rufous-nosed rat (*Oenomys hypoxanthus*) [20] African hedgehogs (*Atelerix* spp.) [59] Four-toed elephant shrew (*Petrodromus tetradactylus*) [60] Red-legged sun squirrel (Heliosciurus rufobrachium) [61] Congo rope squirrel (*Funisciurus congicus*) [62] Thomas’s rope squirrel (*Funisciurus anerythrus*) [63] Unstriped ground squirrel (*Xerus rutilus*) [64] Black-tailed prairie dog (*Cynomys ludovicianus*) [59] Long-tailed chinchilla (*Chinchilla lanigera*) [59] Groundhog (*Marmota monax*) [59] African brush-tailed porcupine (*Atherurus africanus*) [65] Giant anteater (*Myrmecophaga tridactyla*) [66] Gray short-tailed opossum (*Monodelphis domestica*) [59] Common opossum (*Didelphis marsupialis*) [59] Wild boar (*Sus scrofa*) [67] Sooty mangabey (*Cercocebus atys*) [68] Colobus monkey (*Colobus* spp.) [69] Red-tailed monkey (*Cercopithecus ascanius*) [27] Grivet (*Cercopithecus aethiops*) [70] Lesser spot-nosed monkey (*Cercopithecus petaurista*) [70] Allen’s swamp monkey (*Allenopithecus nigroviridis*) [27] Rhesus macaque (*Macaca mulatta*) [66] Cynomolgus macaque (*Macaca fascicularis*) [70] Bornean orangutan (*Pongo pygmaeus*) [66] Chimpanzee (*Pan troglodytes*) [71] Gorilla (*Gorilla* spp.) [66] Human (*Homo sapien*) [72]
Raccoonpox		Raccoon (*Procyon lotor*) [73]
Skunkpox ^1^		Skunk (family *Mephitidae*) [74]
Taterapox		Kemp’s gerbil (*Gerbilliscus kempi*) [75]
Uasin Gishu disease ^1^		Horse (*Equus* sp.) [76]
Vaccinia	Ankara Copenhagen Lister Western Reserve	Laboratory strains
	Brazilian Vaccinia virus	House mouse (*Mus musculus*) [77] Vesper mouse (*Calomys* spp.) [16] South American grass mouse (*Akodon* spp.) [16] Hairy-tailed bolo mouse (*Necromys lasiurus*) [16] Agouti (genus *Dasyprocta*) [14] Atlantic Forest nectomy (*Nectomys squamipes*) [16] Black-footed colilargo (*Oligoryzomys nigripes*) [16] Flavescent colilargo (*Oligoryzomys flavescens*) [16] Terraced rice rat (*Cerradomys subflavus*) [53] Black rat (*Rattus rattus*) [53] Rat-headed rice rats (*Sooretamys angouya*) [16] Hairy Atlantic spiny rat (*Trinomys setosus*) [53] Capybara (*Hydrochoerus hydrochaeris*) [78] Big-eared opossum (*Didelphis aurita*) [53] White-eared opossum (*Didelphis albiventris*) [13] Common opossum (*Didelphis marsupialis*) [13] Bare-tailed woolly opossum (*Caluronys philander*) [53] South American coati (*Nasua nasua*) [15] Six-banded armadillo (*Euphractus sexcintus*) [53] Cat (*Felis catus*) [79] Dog (*Canis familiaris*) [15] Cattle (*Bos* spp.) [80] Horses (*Equus* spp.) [81] Tufted capuchin (*Sapajus apella*) [14] Black howler (*Alouatta caraya*) [14] Human (*Homo sapien*) [80]
	Buffalopox virus	Buffalo (*Bubalus* spp.) [82] Cattle (*Bos* spp.) [83] Human (*Homo sapien*) [83]
	Cantagalo virus	Cattle (*Bos* spp.) [80] Human (*Homo sapien*) [80]
	Horsepox virus	Horse (*Equus* spp.) [29] Cattle (*Bos* spp.) [84] Human (*Homo sapien*) [84]
	Rabbitpox virus Utrecht	European rabbit (*Oryctolagus cuniculus*) [85]
Variola	Variola major Variola minor	Human (*Homo sapien*) [86]
Volepox		California vole (*Microtus californicus*) [87] Pinyon mouse (*Peromyscus truei*) [88]

^1^ Orthopoxvirus-like. Not recognized as a separate species by the International Committee on the Taxonomy of Viruses.

**Table 2:** Comparison of progression of Orthopoxviral diseases in key species.

Virus	Host	Pathology			
		Incubation Period	Classic Site of Skin Lesions	Additional Signs of Disease	Other Notes
Vaccinia	Human [31,33,45,47]	3–5 days	Hands, arms. Can become generalized.	Fever, lymphadenopathy	Duration 4–6 weeks
	Rodent [16,32,36,37]	-	Many species asymptomatic	-	-
	Cattle [31]	1–7 days	Udders, teats	Mastitis	Suckling calves develop oral lesions
	Buffalo [99,100]	-	Udders, teats, ears, eyelids	Mastitis	-
Monkeypox—West African (USA)	Human [66,71,92,96]	4–21 days	Possibly variable: asymptomatic to generalized. Commonly oropharyngeal and genital area.	Fever, lymphadenopathy, fatality rate ~3%	Duration 4 weeks
	Prairie dog [131]	13–24 days	Generalized	Weight loss, fatality in some animals	Duration 4 weeks
Monkeypox—Congo Basin (Zaire)	Human [115,116,120,121,122,124]	4–21 days	Possibly variable: asymptomatic to generalized. Commonly oropharyngeal.	Fever, lymphadenopathy, fatality rate ~10%	Duration 4 weeks
	Thomas’s rope squirrel [63,133]	6–8 days	Ocular, oral. Can become generalized.	Respiratory distress, hepatic necrosis, fatality rate ~ 75%	Virus shed up to 25 days
	Chimpanzee [125]	-	Generalized	Cough, respiratory distress, fatality in infants	Virus in feces, urine, and discarded food
Cowpox	Human [174,181]		Localized. Can become generalized.	Fever, lymphadenopathy	Duration 2–4 weeks
	Rat [162,163]	Up to 11 days	Generalized.	Respiratory distress	Virus shed up to 21 days.
	Cat [149,166]	-	Head, neck, forelimbs	Lymphadenopathy, fatality in immunosuppressed	Duration 4–5 weeks
	Cattle [170]	3–7 days	Udders, teats. Can become generalized.	Fever, mastitis	Duration 4 weeks, suckling calves develop oral lesions
Camelpox	Human [37,177]	7–10 days	Hands.	Fever, itching	Duration 4–6 weeks
	Camel [37,177]	-	Head, neck, inguinal area	Fever, nasal discharge, respiratory distress, gastrointestinal upset, fatality rate up to 25%	-

## Data Availability

Not applicable.
